# Prokaryotic Na^+^/H^+^ Exchangers—Transport Mechanism and Essential Residues

**DOI:** 10.3390/ijms23169156

**Published:** 2022-08-15

**Authors:** Miyer Patiño-Ruiz, Constanța Ganea, Octavian Călinescu

**Affiliations:** 1 Department of Biochemistry, Groningen Biomolecular Sciences and Biotechnology Institute and Zernike Institute for Advanced Materials, University of Groningen, Nijenborgh 4, 9747 AG Groningen, The Netherlands; 2 Department of Biophysics, Faculty of Medicine, “Carol Davila” University of Medicine and Pharmacy, Bd. Eroii Sanitari 8, 050474 Bucharest, Romania

**Keywords:** Na^+^/H^+^ exchange, transmembrane transport, cation proton antiport, NhaA, NhaP, NhaB, NhaC, NhaD

## Abstract

Na^+^/H^+^ exchangers are essential for Na^+^ and pH homeostasis in all organisms. Human Na^+^/H^+^ exchangers are of high medical interest, and insights into their structure and function are aided by the investigation of prokaryotic homologues. Most prokaryotic Na^+^/H^+^ exchangers belong to either the Cation/Proton Antiporter (CPA) superfamily, the Ion Transport (IT) superfamily, or the Na^+^-translocating Mrp transporter superfamily. Several structures have been solved so far for CPA and Mrp members, but none for the IT members. NhaA from *E. coli* has served as the prototype of Na^+^/H^+^ exchangers due to the high amount of structural and functional data available. Recent structures from other CPA exchangers, together with diverse functional information, have allowed elucidation of some common working principles shared by Na^+^/H^+^ exchangers from different families, such as the type of residues involved in the substrate binding and even a simple mechanism sufficient to explain the pH regulation in the CPA and IT superfamilies. Here, we review several aspects of prokaryotic Na^+^/H^+^ exchanger structure and function, discussing the similarities and differences between different transporters, with a focus on the CPA and IT exchangers. We also discuss the proposed transport mechanisms for Na^+^/H^+^ exchangers that explain their highly pH-regulated activity profile.

## 1. Introduction

An essential component of homeostasis for every organism is the control of the intracellular concentrations of cations, with Na^+^, K^+^, Ca^2+^, and H^+^ being of particular interest [1,2,3,4,5,6]. The mechanisms for regulating the concentrations of these ions are diverse, but they all rely on the existence of one or several active transport systems that can remove excessive amounts of the respective ion from the cell if the concentration ever gets too high. This can be performed by pumps that directly consume ATP (such as the Na^+^/K^+^, H^+^/K^+^ or Ca^2+^-ATP-ases) or by secondary active transporters that use the electrochemical gradient of one cation to transport another cation against its gradient (such as Na^+^/Ca^2+^ exchangers that remove excessive Ca^2+^ from the cell relying on a pre-existing Na^+^ gradient). Na^+^/H^+^ exchangers fall into this second category—they are secondary active transporters present in all organisms that remove excess Na^+^ or H^+^ from the cell or certain organelles, using, as driving force, an inward directed gradient of the opposite ion [7,8,9].

13 Na^+^/H^+^ exchanger-encoding genes have been identified in the human genome. The most investigated human Na^+^/H^+^ exchangers are the nine isoforms of the NHE family [9,10,11]. The NHE isoforms expressed mainly in the plasma membrane (NHE1–5) are the best understood, and have the export of H^+^ from the cell as a main role, using the transmembrane Na^+^ gradient as a driving force [9]. Comparatively, the functions of the predominantly intracellular membrane NHEs (NHE6–9) have not been fully elucidated. Though it was shown that these contribute to the pH regulation of intracellular compartments, it has been speculated that at least some of these isoforms might also catalyze K^+^/H^+^ exchange [7,9]. Deficiency in the human NHE-type exchangers has been associated with a wide variety of conditions, including hypertension, ischemia, epilepsy, or cancer [9,10]. Besides the well-characterized NHE exchangers, two Na^+^/H^+^ exchangers which share a higher homology to bacterial NhaA proteins were identified and accordingly named as NHA1 and NHA2 [11]. The NHA1 human transporter is expressed in testes and has a role in sperm motility, with mice deficient in the *Nha1* gene displaying reduced fertility [12]. The NHA2 exchanger similarly has a role in sperm motility [12], and has additionally been shown to aid Na^+^ reabsorption in the kidney [13] and facilitate insulin secretion by β-cells [14]. Finally, two more human Na^+^/H^+^ exchangers, SLC9C1 and SLC9C2, have been identified, with the former being sperm-specific [15], whereas the latter has, so far, not been functionally characterized [9]. Extensive reviews on the impact of Na^+^/H^+^ exchangers of the NHE and NHA families on human health can be found in the literature [9,10,11,16,17,18].

Though the human Na^+^/H^+^ exchangers are of paramount interest due to their direct medical importance, the investigation of prokaryotic Na^+^/H^+^ exchangers is equally valuable, as they can serve as model systems for their human homologues [19,20], as well as being important in bacterial survival and virulence [1,3,4,21]. NhaA from *E. coli* (EcNhaA), which is essential in the survival of the bacterium under conditions of high salinity [22], is the most studied Na^+^/H^+^ exchanger [20], as it belongs to the same transporter superfamily as the human NHE and NHA exchangers [23]. The structure of EcNhaA was the first structure of a Na^+^/H^+^ exchanger to be solved, in 2005 [24], joined later by structures of prokaryotic exchangers from the NapA [25] and NhaP families [26,27], and, more recently, of the human NHE1 [28] and mammalian NHA2 [29]. However, as the resolution of all these structures is limited in the range of 3–4 Å, several questions, including the exact location of the substrate binding site(s), still remain unanswered.

So far, reviews on prokaryotic Na^+^/H^+^ exchangers have focused mainly on EcNhaA and closely related transporters from the Monovalent Cation: Proton Antiporter superfamily (CPA) [8,20,30], despite the fact that other, unrelated prokaryotic Na^+^/H^+^ exchangers also exist, as we describe in the following section. The aim of the current review will be the discussion of the known aspects of prokaryotic Na^+^/H^+^ exchanger structure and function, including available information on transporters from the CPA superfamily, but also the Ion Transporter (IT) superfamily and the Na^+^-transporting Multiple Resistance and pH (Mrp) superfamily, which share no sequence or structural similarities with CPA exchangers. In particular, the focus will be on the proposed models of the transport mechanism, as well as the amino acid residues that have been shown to contribute to functionality and substrate binding in the CPA and IT transporter superfamilies.

## 2. Classification

Several efforts to design a systematic classification of membrane transporters have produced different nomenclatures and databases; some of them are general, and some are restricted to a group of organisms or substrates [31]. In the classification of human genes maintained by the HUGO Gene Nomenclature Committee (HGNC) [32], Na^+^/H^+^ exchangers belong to the series of solute carrier proteins (SLC) within the SLC9 family [11]. The SLC family is further subdivided into three subfamilies: SLC9A, from which there are nine paralogs in the human genome (SLC9A1–9) encoding NHE1–9; the SLC9B subfamily, which includes SLC9B1 and SLC9B2, encoding NHA1 and NHA2, and SLC9C, with two members, SLC9C1 and SLC9C2 [11].

A broader classification of membrane transporters, the Transporter Classification system (TC), includes membrane proteins from all organisms. In accordance with the TC system, the Transporter Classification Database (TCDB) [31,33,34,35] is maintained, which, at this moment, consists of more than 20,000 proteins. Every transporter has a TC identification number, which contains information about the class of transporter, subclass, family, subfamily, and the transported substrate or range of substrates. As it is a universal classification, the TC system includes the SLC members, which can be found distributed within different superfamilies and families. Due to the focus of this review on prokaryotic Na^+^/H^+^ exchangers, some of which have no homologues in the SLC series, in the following, we will employ the TC system for classification.

Na^+^/H^+^ exchangers are distributed within several superfamilies according to the TCDB [33,34,35]: the CPA superfamily, the IT superfamily, and the Mrp superfamily. Human Na^+^/H^+^ exchangers (SLC9 members according to HGNC) all belong to the CPA superfamily. Recently, a Na^+^/H^+^ exchanger consisting of only 103 amino acids and three transmembrane helices (TMHs) has been identified [36]. As it is the smallest of the Na^+^/H^+^ exchangers discovered so far, it has been denoted as NhaM (from minimal). Based on phylogenetic analysis, it has not been classified within any of the other families, and has been proposed to belong to a separate NhaM family [36].

### 2.1. The CPA Superfamily

The majority of identified and studied Na^+^/H^+^ exchangers belong to the CPA superfamily. Members of this superfamily share a common transmembrane organization of 10–14 TMHs with detectable sequence similarity [23]. Entries assigned to the CPA superfamily have been recognized to have the Na_H_Exchanger motif, Pfam00999, in their sequence, according to the Conserved Domain Database [37]. Out of the subfamilies of the CPA superfamily, three contain Na^+^/H^+^ exchangers: CPA1 (TC identifier: 2.A.36), CPA2 (2.A.37), and the small family of Na^+^-transporting carboxylic acid decarboxylase (NaT-DC) (3.B.1) [35].

Proteins that constitute the CPA1 family are derived from Gram-positive and Gram-negative bacteria, blue-green bacteria, archaea, yeast, plants, and animals. Members of this family are known to transport Na^+^ or Li^+^ in exchange for H^+^ in an electroneutral and pH-dependent manner [23]. The CPA1 family includes the prokaryotic NhaP transporters, human NHEs, as well as plant NHXs [23,38]. From its prokaryotic members, the best studied are two NhaP exchangers, the structures of which have been solved: NhaP1 from *M. jannaschii* (MjNhaP1) [26], and NhaP from *P. abyssi* (PaNhaP) [27]. The NhaP family also contains a subfamily, designated NhaP-II, in which some K^+^-specific transporters are found [38], such as the NhaP transporters from *V. cholerae* [39].

Members of the CPA2 family consist of between 300–900 amino acids; have 10–14 TMHs; and are distributed in bacteria, archaea, and eukaryotes. This family is subdivided into two subfamilies: CHX and NHA. Within the CHX clade, plant and fungal transporters may be found, such as the *CHX* genes from *A. thaliana* and the *KHA* genes from *S. cerevisiae* [20]. A group of related genes in animals has been identified and named as *NHA*, on the basis of their similarity to fungal *NHA* and bacterial *nhaA* genes [40]. The prototype of the CPA2 family, and the CPA superfamily in general, is the bacterial EcNhaA, an electrogenic transporter with a H^+^:Na^+^ stoichiometry of 2:1 [41].

Finally, members of the NaT-DC family catalyze the decarboxylation of a substrate carboxylic acid, and use the energy released to drive the extrusion of one or two sodium ions from the cytoplasm. For this family, only prokaryotic members have been identified [23]. The ß-subunits of the multimeric proteins that belong to this family possess nine TMHs, and show sufficient sequence similarity to the Na^+^/H^+^ antiporters of the CPA2 family.

### 2.2. The IT Superfamily

Eighteen different families of transporters have been classified as members of the IT superfamily. Besides sharing some motifs, what all these families have in common is that the substrates for the transporters are charged organic or inorganic chemical species, either anionic or cationic [42]. Four different families of Na^+^/H^+^ exchangers can be found within this superfamily: NhaB (TC identifier: 2.A.34), NhaC (2.A.35), NhaD (2.A.62), and NhaE (2.A.111), named alphabetically in the order of their discovery. We will consider each of these in brief.

The best studied of these are the NhaB family exchangers, which consist of 9–12 TMHs with a size range of 500–550 amino acids. NhaB was first identified in *E.* coli, where it complements the activity of EcNhaA [43,44], but it is expressed in other Gram-negative bacteria as well, including *V. cholerae, V. alginolyticus, or K. pneumoniae*. The stoichiometry of NhaB from *E. coli* was determined to be 3:2 H^+^:Na^+^ [45].

Genes encoding NhaC proteins have been identified in the Gram-positive *B. firmus*, *B. subtilis*; the Gram-negative *H. influenziae*, *Vibrio cholerae*; and the archaea *P. furiosus* [46,47]. NhaC from *B. firmus* has a putative size of 462 amino acids and 12 TMHs, and its role as a Na^+^/H^+^ antiporter in *B. firmus* OF4 was confirmed from complementation of a salt-sensitive strain of *E. coli*, as well as from the detection of electrogenic ^22^Na^+^ extrusion in membrane vesicles from the parental strain [46]. 

Sequences from the NhaD family have low identities with the Na^+^/H^+^ antiporters from the CPA2 and NhaB families. The first member of this family was identified in the halophilic marine bacterium, *V. parahaemolyticus* [48]. This sequence was expressed in a salt-sensitive strain of *E. coli*, and the Na^+^(Li^+^)/H^+^ antiport activity was measured in membrane vesicles of the transformed strain. NhaD has been suggested to be a marker of virulence in organisms of the genus, *Vibrio*; although, this has not been experimentally confirmed [49]. As NhaD transporters have been identified in several marine halophilic bacteria, this indicates NhaD as an important player in salt tolerance in the pH range of 7 to 9 [50].

The existence of a separate NhaE family of Na^+^/H^+^ exchangers was proposed following studies on a Na^+^/H^+^ exchanger from the thermohalophilic bacterium, *R. marinus* [51]. Heterologous expression of this sequence in *E. coli* confers resistance to high concentrations of Na^+^, but not Li^+^ and at low pHs (6.5–7.5). In line with this, residues presumably participating in Li^+^ coordination in members of the NhaD family were not found in this transporter [51]. A homologous protein was identified and characterized in the pathogenic bacteria *N. meningiditis*. Unlike NhaE from *R. marinus*, NhaE from *N. meningiditis* restored the salt resistance to a salt-sensitive strain of *E. coli* at high concentrations of Na^+^ and Li^+^, and in a broader range of pH 6.5–8.5, with an optimal activity around pH 7 [52].

### 2.3. The Na^+^-Transporting Mrp Superfamily

Members of the Mrp superfamily are multi-subunit membrane proteins distributed within the archaeal and bacterial kingdoms. The Mrp superfamily is subclassified into four families containing transporters of different functions: two families contain transporters with dehydrogenase activity, one family consists of oxidases, and one family consists of proteins with cation/H^+^ antiporter activity. Despite this latter family not being part of the CPA superfamily, it has been designated as CPA3 (TC identifier: 2.A.63). Members of the dehydrogenase and oxidase families exert their transport and redox function as active participants in the electron transfer chain, whereas the main role of the CPA3 family is related to Na^+^ and H^+^ homeostasis [34].

The CPA3 family contains Mrp secondary transporters that mainly work as antiporters of Na^+^ (or K^+^) for H^+^. Though cation/proton antiporters from other families typically consist of one subunit assembled in the membrane as an oligomer, members of this family are assembled from 6–7 different subunits that form a complex of multiple membrane proteins [53].

A Na^+^/H^+^ exchanging Mrp was first discovered from the rescue of growth at alkaline conditions of an alkaline-sensitive strain of *B. halodurans* by complementation with a DNA fragment containing several open reading frames [54]. Proteins encoded by the identified ORFs were highly hydrophobic, indicating their character as membrane proteins, and presented similarities with ion-translocating electron transport complexes [55]. Furthermore, vesicles from the transformed strain showed recovery of the membrane-potential-driven Na^+^/H^+^ antiport activity [54,55]. Since then, Mrp has been confirmed in other Gram-positive bacteria (*B. subtilis*), Gram-negative bacteria (*V. cholerae*, *S. meliolitis*, *P. aeruginosa*, *A. tumefaciens*), and archaea (*M. acetivorans*) [53].

## 3. Physiological Role

Most prokaryotic Na^+^/H^+^ exchangers that have been investigated so far have been found to regulate the salt resistance of their host organisms. In *E. coli*, this is done mainly by the NhaA and NhaB transporters [43,44,56,57]. The deletion of both the *nhaA* and *nhaB* genes prevents the growth of *E. coli* at even moderate Na^+^ concentrations [44], but does not prevent growth at low Na^+^ concentrations, where Na^+^ export can be performed by other transport systems, mainly the Ca^2+^/H^+^ exchanger, ChaA [3]. As both NhaA and NhaB are electrogenic, with a 2:1 and 3:2 H^+^:Na^+^ stoichiometry, respectively [45,58], the negative-inside membrane potential can drive Na^+^ extrusion as well [3], which is particularly important when the extracellular pH is alkaline. Consequently, this difference in stoichiometry might explain why NhaA supports the growth of *E. coli* at alkaline pH, whereas NhaB does not [3]. Under low Na^+^ conditions, only NhaB is expressed, whereas the presence of Na^+^ or Li^+^ induces the expression of NhaA [3,59].

In *V. cholerae*, several Na^+^/H^+^ exchangers are expressed, belonging to the NhaA, NhaB, NhaD, and NhaP families [39,60]. In contrast to *E. coli*, the deletion of Na^+^/H^+^ exchanger genes has a smaller effect on the Na^+^ resistance profile of *V. cholerae*. Thus, the deletion of any of the *nhaA*, *nhaB*, or *nhaD* genes alone had no effect on the growth profile of the bacterium at high Na^+^ concentrations [60], though mutants lacking the *nhaA* gene were growth-impaired in the presence of Li^+^ at high pH. Even a triple mutant strain of *V. cholerae* lacking *nhaA*, *nhaB*, and *nhaD* could still survive in the presence of high Na^+^ (but not Li^+^) concentrations, as Na^+^ export can be performed in *V. cholerae* by the redox-driven sodium pump, NQR [60]. Three NhaP family exchangers are expressed in *V. cholerae*, VcNhaP1, VcNhaP2, and VcNhaP3 [39]. None of these are able to catalyze Li^+^/H^+^ exchange, but all are capable of transporting K^+^, with VcNhaP3 being exclusively a K^+^/H^+^ antiporter [39]. The deletion of all NhaP genes of *V. cholerae* significantly impaired growth of the bacterium in the presence of K^+^ [39].

Similar to the findings in *V. cholerae*, in the halophile, *V. alginolyticus*, the NhaA, NhaB, and NhaD Na^+^/H^+^ exchangers have an important role in Na^+^ and Li^+^ tolerance, with the deletion of all three genes encoding for these transporters impairing growth in the presence of Li^+^ and at high Na^+^ concentrations [61]. As in *V. cholerae*, the survival of this triple deletion mutant in the presence of Na^+^ could be explained by Na^+^ extrusion through a respiratory Na^+^ pump [61]. The triple deletion mutant of *V. alginolyticus* was also found to be growth impaired under hypotonic conditions, suggesting that Na^+^/H^+^ antiport plays a role in osmoregulation [61].

In *P. aeruginosa*, four Na^+^/H^+^ exchangers are present, NhaP, NhaP2, NhaB, and Mrp [62,63,64]. The deletion of the four genes encoding for these exchangers still allowed the growth of *P. aeruginosa* in the presence of Na^+^ under some conditions, but always slower and to lower final cell densities than the WT strain [62]. Additionally, the deletion of the four Na^+^/H^+^ antiport genes caused a decrease in the production of pyocyanin and an increase in biofilm formation and glycerol metabolism [62].

In *Y. pestis*, the NhaA and NhaB proteins have been shown to be essential for virulence [21]. Thus, a strain deficient in the *nhaA* and *nhaB* genes was not lethal to mice in a bubonic plague mouse model, and its growth was strongly inhibited in sheep blood and serum, showing that NhaA and NhaB of *Y. pestis* are essential for surviving Na^+^ toxicity in blood [21].

As with NhaB, NhaC Na^+^/H^+^ exchangers have also been shown to have Na^+^ or Li^+^ extrusion as a main role. Thus, NhaC from *Bacillus sp*. G1 seems to play a role as a Na^+^ extrusion system at pH 7.5, whereas at pH 10, it ensures pH homeostasis in the presence of low Na^+^ concentrations [65]. This latter activity suggests its electrogenic character as a Na^+^/H^+^ antiporter. Interestingly, one of the two homologues of NhaC in *B. subtilis*, YqkI, acts as a malic acid/sodium lactate antiporter in an electroneutral manner, whereas the other homologue, YheL, works as an electrogenic Na^+^/H^+^ exchanger [66]. 

Mrp Na^+^/H^+^ exchangers have been shown to catalyze Na^+^ (but also Li^+^ or K^+^, depending on organism) export, as well as bile acid export [67]. Beside these roles, Mrp transporters were shown to be contributors to sporulation in *B. subtilis* [68], and to facilitate the virulence of *P. aeruginosa* [69].

EcNhaA has been shown to be symmetrical with respect to its function, being able to function equally well at zero potential both in its physiological mode (Na^+^ export), as well in the reverse mode (H^+^ export) [70]. It is the membrane potential that ensures that transport mainly occurs in the physiological direction [71]. It stands to reason then that an electroneutral Na^+^/H^+^ exchanger would not have that limitation, and would also be able to have the export of H^+^ as a main role. Indeed, some prokaryotic Na^+^/H^+^ exchangers, such as MjNhaP1 or PaNhaP, are believed to function mainly as H^+^ export systems [72,73]. In this role, the exchanger would export H^+^ when their intracellular concentration becomes too high, ensuring pH homeostasis. To prevent the cytoplasm from becoming too alkaline, transport would need to cease if the pH rises too far above the physiological level. An explanation of how this regulation can occur is provided by the competition model of transport, discussed in Section 6 and, in more detail, reviewed in one of our previous papers [71].

A summary of the physiological role of selected Na^+^/H^+^ exchangers from different families is presented in Table 1.

## 4. Structural Particularities

### 4.1. The NhaA Fold

**Figure 1:**
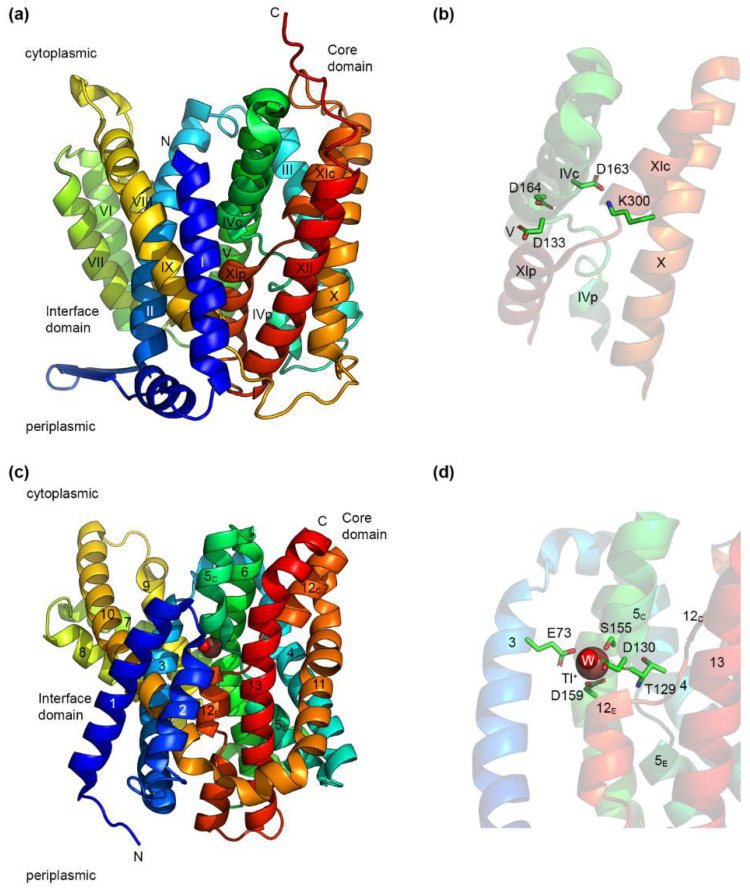
Structures of EcNhaA and PaNhaP. (**a**) Crystal structure of EcNhaA (PDB accession number: 4ATV) obtained at low pH in the inward-open conformation. TMHs are labeled according to the original structure publication [24]. (**b**) A detailed look at the structure in panel (**a**) showing the crossed interrupted helices of EcNhaA and its putative substrate binding region. Three aspartate residues are present in this region, out of which, Asp163 forms a salt bridge with Lys300. (**c**) The crystal structure of PaNhaP (PDB accession number: 4CZA) obtained at high pH in the inward-open conformation. A Tl^+^ ion (also a substrate of PaNhaP) and a water molecule are present in the substrate binding site. TMHs are labeled according to the original structure publication [27]. (**d**) A detailed look at the structure in panel (**c**), showing the substrate binding site of PaNhaP and the residues coordinating the Tl^+^ ion. Structures were drawn using the PyMOL Molecular Graphics System, Version 2.6.0a0, Schrödinger, LLC.

The only structures of prokaryotic Na^+^/H^+^ exchangers that have been solved so far are all of transporters that belong to the CPA superfamily. Thus, structures have been published for the electrogenic EcNhaA (Figure 1a) [24,79] and NapA from *T. thermophilus* (TtNapA) [25], which belong to the CPA2 family. Additionally, structures for the NhaA exchanger from *S. typhimurium* were recently made available in the Protein Data Bank [80] (PDB accession numbers: 7A0W, 7A0Y, 7A0X). From the CPA1 family, the structures of two electroneutral Na^+^/H^+^ exchangers, MjNhaP1 [26] and PaNhaP [27], were published to date. 

Despite differences in the number of TMHs, all of these structures of CPA Na^+^/H^+^ exchangers share a similar fold, called the NhaA fold [41]. This fold is characterized by the presence of inverted topology repeats forming two domains—a core domain responsible for ion transport, and a dimerization domain. As a further particularity, the core domain contains two partially unwound TMHs, where the putative Na^+^ binding site is located (Figure 1b). It should be noted that the NHA fold is not restricted to Na^+^/H^+^ exchangers, as the same fold has been identified in the structure of two apical sodium-dependent bile acid transporters (ASBT), from *N. meningitidis* [81] and *Y. frederiksenii* [82].

As the most studied Na^+^/H^+^ exchanger, EcNhaA is the prototype of the CPA Na^+^/H^+^ exchangers [20,41,83]. In its physiological environment, EcNhaA is a dimer [79], but it is functional as well in the monomeric form [84,85], with dimerization providing enhanced stability [85,86]. The EcNhaA monomer (Figure 1a) is built up of 12 TMHs divided into two domains. The dimerization domain consists of TMHs I, II, VI, VII, VIII, and IX, and a β-hairpin at the periplasmic side, between TMHs I and II. Both the β-hairpin and the assembly of TMHs VI and VII have been found to be essential for the correct assembly of the dimer [85]. The core domain responsible for ion transport consists of a six-helix bundle formed by TMHs III, IV, and V, and the topologically inverted TMHs X, XI, and XII [27]. TMHs IV and XI are interrupted in the middle by an unwound region, and cross each other in the middle of the membrane. Each of their halves is denoted as either p (periplasmic) or c (cytoplasmic), depending on their position (Figure 1a). The structures of EcNhaA solved so far [24,79] are open towards the cytoplasm. TMHs II, IVc, IX, and V form a large cytoplasmic funnel lined by negatively charged residues allowing the access of the substrate ions towards the interior of the membrane [24]. By comparison, the periplasmic funnel formed by TMHs II, VIII, and XIp is shallower.

The resolution (and likely, the low pH) at which the structures of EcNhaA were determined did not allow identification of a Na^+^ ion in the substrate binding site. However, mutational analysis and computational studies have revealed a series of charged residues located at the middle of the core domain that are likely to be part of the Na^+^ and H^+^ binding sites [79,83]. Figure 1b shows the residues, Asp 133, Asp163, and Asp 164, as well as the residue, Lys 300, which forms a salt bridge with Asp 163. The roles of these residues will be discussed in more detail in Section 5. 

Figure 1c shows the crystal structure of another CPA Na^+^/H^+^ exchanger, PaNhaP. PaNhaP is also a dimer under physiological conditions, but has an additional TMH at the N-terminus that is not present in EcNhaA [27]. The dimerization domain consists of TMHs 1, 2, 3, 7, 8, 9, and 10, whereas the core domain is formed, as in EcNhaA, of a six-helix bundle (TMHs 4, 5, 6, 11, 12, and 13). A six-helix inverted repeat is formed by TMHs 1–6 and 8–13, with TMH 7 in the middle. The same motif of two crossed interrupted helices is present in PaNhaP as well, TMHs V and XII, which are denoted, according to [27], in two halves—extracellular (E) and cytoplasmic (C). The structure of PaNhaP is missing the β-hairpin of EcNhaA, with the monomers held together by ion bridges at pH 8 [27]. Structures were determined for PaNhaP in acidic (pH 4) and alkaline (pH 8) environments, both in the cytoplasmic open conformation, with the major differences between the two structures occurring at the dimer interface [27]. Unlike EcNhaA, where the substrate ion could not be detected in the structure, the crystal structure of PaNhaP at pH 8 shows the presence of the ion Tl^+^ (Figure 1d), which is able to replace Na^+^ and bind to the exchanger [27,73]. In the structure, the Tl^+^ ion is coordinated directly by the side chains of three amino acid residues, Glu73, Asp159, and Ser155; the main chain carbonyl group of Thr129; and by a water molecule forming a hydrogen bond with Asp130 [27].

A periplasmic-open structure was obtained for TtNapA [25]. Despite the low sequence homology to EcNhaA, the structures are highly similar [25], with the main difference lying in the relative position of the core and dimerization domains. This has led to the proposal of a two-domain elevator mechanism for the translocation of substrates in CPA Na^+^/H^+^ exchangers [25,28]. However, the actual switch between the two conformations is likely to function by a combination of the rocking bundle and elevator mechanism, as evidenced by recent studies [87,88].

What are the evolutionary origins of the NhaA fold? A recent study [36] might shed some light on this question through the identification of the NhaM Na^+^/H^+^ exchanger. NhaM possesses only three TMHs, out of which TMH 1 contains its sole indispensable negatively charged residue, Glu15, as well as a region predicted to be unwound. TMH 2 of NhaM contains a conserved His residue (H43) that was shown to be essential for correct function of the transporter, and was proposed to be analogous to the Lys300 residue of EcNhaA. Interestingly, NhaM cannot function in its monomeric form [36], presumably due to the too low number of TMHs preventing the formation of a functional core domain for the coordination of the transported Na^+^ ions. Instead, NhaM is only functional when forming homo-oligomers of various sizes [36]. Whether these assembled oligomers finally resemble an NhaA-fold exchanger will have to be revealed by future structural studies.

### 4.2. IT Na^+^/H^+^ Exchangers

As discussed previously, not all Na^+^/H^+^ exchangers belong to the CPA superfamily. Is the NhaA fold also found in the IT superfamily Na^+^/H^+^ exchangers? Very likely not. Though no structures are available for the IT Na^+^/H^+^ exchangers, we decided to use the AlphaFold2 neural network [89,90] to model the structure of NhaB from *Klebsiella pneumoniae* (KpNhaB), a Na^+^/H^+^ exchanger that we recently studied [91,92]. AlphaFold2 is an artificial-intelligence-based system developed by DeepMind that was shown, in 2020, to provide the best accuracy in the predicted protein structures compared to its competitors [93]. In the last two years, it has been used to build a database of protein structures [90], which now contains over 200 million entries, including predicted structures for the majority of proteins in the UniProt protein database [94].

**Figure 2:**
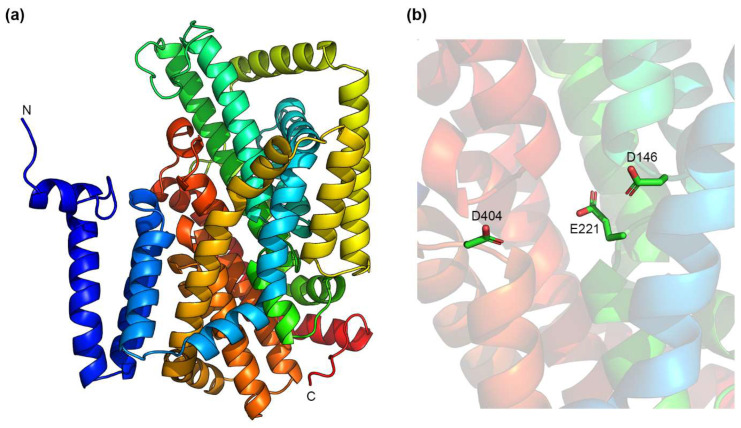
Predicted structure of KpNhaB using the AlphaFold2 neural network [89,90]. (**a**) A side view of the predicted structure. (**b**) The interior cavity of KpNhaB contains three negatively charged residues that are predicted to be found in helical hairpin regions. Structures were drawn using the PyMOL Molecular Graphics System, Version 2.6.0a0, Schrödinger, LLC.

The resulting model structure (Figure 2a) is dissimilar to the structures of CPA Na^+^/H^+^ exchangers shown in Figure 1, and rather resembles the structure of the Na^+^/dicarboxylate transporter, INDY, from *V. cholerae* (VcINDY), another member of the IT family [95]. This is consistent with the recent finding that the predicted structure (using AlphaFold2 as well) of NhaD1 from *H. hydrothermalis* Y2 (HhNhaD1) also resembles that of VcINDY [96]. As for VcINDY, the predicted structure of KpNhaB contains a series of helical hairpins towards the middle of the membrane (Figure 2a). In these hairpins, several negatively charged residues are present (Figure 2b), including Asp146 and Asp404, which we found to be essential for the function and stoichiometry of KpNhaB [92]. Unlike CPA exchangers, which have a Lys or Arg present close to the substrate binding site, we could not identify any such residues in the vicinity of the hairpin regions in the predicted KpNhaB structure.

### 4.3. Mrp Na^+^/H^+^ Exchangers

Most of the functional and structural information for the Mrp complex has been obtained from studies on the purified and reconstituted Mrp antiporter from *B. pseudofirmus* OF4 [97], and from the cryo-EM structures of the *A. flavithermus* homologue [98] and the Mrp complex from *Dietzia* sp. DQ12-45-1b [99]. Mrp complexes are assembled from seven subunits, named as A, B, C, D, E, F, and G, all of them integral membrane proteins with sizes between 10 and 90 kDa. Altogether, these form the MrpABCDEFG complex, with a size of about 220 kDa, which is organized as a 440 kDa dimer. All the subunits are needed to have full Na^+^/H^+^ antiport activity, but in *B. subtillis*, MrpE seems to be dispensable for ion transport activity [100]. Furthermore, structural data indicate that MrpE is involved in dimerization. MrpD and MrpA are direct participants in the cation and H^+^ translocation, whereas the other subunits are believed to play roles in assembly and stability [98]. 

As Mrp is a multi-subunit Na^+^/H^+^ exchanger, its transport mechanism and the pathways that Na^+^ and H^+^ follow through it to get to the other side of the membrane are highly different and more complex than in CPA and IT exchangers. Considering our focus in this review was on the latter two types of Na^+^/H^+^ exchangers, we chose not to delve into the intricacies regarding the Mrp structure and transport mechanism, but recommend instead the detailed description by Steiner and Sazanov [98].

Why would Mrp need to complex seven subunits to fulfill the same function that a single polypeptide chain can efficiently sustain? Initially, it was proposed that, in addition to its function as a secondary antiport, a primary function as Na^+^/H^+^ exchange energized by redox energy can take place, but there is no experimental evidence supporting this idea [101]. Another explanation considers the possibility that some subunits might have independent catalytic activities besides Na^+^/H^+^ antiport. This idea has been supported by experimental data suggesting that MrpF of *B. subtilis* acts as a cholate transporter [67,102]. It can be hypothesized that multiple subunits guarantee more efficiency in the capture of H^+^ or Na^+^, particularly in the alkaline conditions in which Mrp is active and exerts its function or can confer versatility in the interaction with other proteins in order to perform complex physiological functions [101].

## 5. Essential Residues and the Substrate Binding Site(s)

The number of mutational studies performed on prokaryotic Na^+^/H^+^ exchangers is extremely large, aided by the ease of expressing these mutants in *E. coli*, which allows for a relatively straightforward assessment of, at least, a few basic aspects (expression level, functionality in providing saline resistance, assaying transport activity in native vesicles). We will focus, in this section, on describing only the most remarkable mutations that alter the activity of Na^+^/H^+^ exchangers, as well as on the features that Na^+^/H^+^ exchangers from different families have in common. For a comprehensive description on the effects of mutations dedicated to EcNhaA alone, a recent review is available [30].

### 5.1. The CPA Motif

A phylogenetic study on more than 6000 CPA members [38] proposed a novel classification of the CPA superfamily, and revealed a motif of eight amino acid residues that could be used to assign CPA Na^+^/H^+^ exchangers to 15 different clades, six for the CPA1 family and nine for the CPA2 family. This motif was denoted [38] as X_1_X_2_X_3_X_4_ … [E/-]_5_ - - - X_6_D_7_ … [R/K]_8_, where the dots represent a large number of residues, and the dashes represent an individual amino acid residue. Figure 1b shows four (positions 3, 6, 7, and 8) of the seven residues of the CPA motif present in EcNhaA, with Ala131, T132, and Ile134 being omitted for clarity.

Using the TMH notations for EcNhaA from Figure 1a, the first four residues are in the interrupted region of TMH IV [38]. These vary significantly throughout the CPA superfamily, and were used to assign transporters into different clades. The glutamate at position 5 locates to TMH V, and is fully conserved in the CPA1 family, but absent in the CPA2 members. This, coupled with the CPA1 specific arginine at position 8, located on TMH X, suggests a potential electrostatic interaction between the two residues [38]. Indeed, these residues form an ion bridge in the MjNhaP1 and PaNhaP structures [26,27].

Positions 6–7 represent the ND or DD motif present on TMH V that was previously considered to separate between electrogenic and electroneutral members of the CPA superfamily [38,103,104,105]. One or both of these residues have invariably been shown to be essential for the function of several CPA Na^+^/H^+^ exchangers [27,103,106]. However, recent studies on the human NHA2 exchanger have shown it to be electroneutral, despite the presence of the same DD motif as in the electrogenic EcNhaA or TtNapA [29]. 

Finally, position 8 is a conserved lysine or arginine residue that was found in the published structures of NhaA fold exchangers to form an ion bridge with either the glutamate at position 5, for the CPA1 members, MjNhaP1 and PaNhaP [26,27], or with the aspartate residue at position 6, in the CPA2 exchangers, EcNhaA and TtNapA [25,79].

### 5.2. The Substrate Binding Site of CPA Na^+^/H^+^ Exchangers

The identification of amino acid residues responsible for coordinating the substrate ions in Na^+^/H^+^ exchangers has been attempted mainly by functional, mutational, and computational studies, with only the PaNhaP structure showing a metal ion (Tl^+^) coordinated in the substrate binding site [27]. In general, Na^+^ coordination in proteins is done by oxygen atoms, either from main chain carbonyls, side chains, or water molecules, with the most common coordination number being six [107]. At the same time, binding a H^+^ ion requires the existence of a single protonatable residue.

Are there different binding sites for Na^+^ and H^+^? Most evidence points towards a negative answer to this question in the case of CPA Na^+^/H^+^ exchangers. Functional analysis of several Na^+^/H^+^ exchangers has shown that Na^+^ and H^+^ compete for a common binding site [70,71,73,91,105,108]. Thus, the protonatable residues that bind H^+^ are also part of the residues that coordinate the Na^+^ ion, such that when Na^+^ binds to the transporter, it can only do so if the respective residues are unprotonated. The reverse is also valid, with H^+^ only able to protonate these residues if Na^+^ is displaced. This competition mechanism will be discussed in more detail in Section 6. A major difference obviously exists between the electroneutral CPA exchangers, such as PaNhaP or MjNhaP1, and the electrogenic EcNhaA or TtNapA, with the latter type needing to accommodate an additional protonatable residue in their binding site.

The location of the substrate binding site in EcNhaA is proposed to be towards the center of the membrane (Figure 1b), in close proximity to the unwound regions of TMHs IV and XI [106]. A large number of mutational studies have been performed on EcNhaA using several types of assays for probing protein function, including fluorescence spectroscopy, survival of bacteria in selective media, solid supported membrane (SSM)-based electrophysiology, calorimetry, or radioligand binding. Aided by computational studies, these have provided enough information to locate the main residues responsible for Na^+^ and H^+^ binding, though some issues still remain under dispute.

#### 5.2.1. Asp164

A conserved aspartate residue on TMV, Asp164, has been shown as indispensable for antiport activity in EcNhaA [106,109], as well virtually all investigated CPA Na^+^/H^+^ exchangers, for example, in NhaA from *H. pylori* [110,111], MjNhaP1 [103], TtNapA [77], or the K^+^-selective NhaP2 from *V. cholerae* [39]. As a protonatable residue, Asp164 has been proposed to be both a coordinating residue for the Na^+^ ion, as well as the binding residue for one of the two H^+^ transported by EcNhaA, and this has been supported by a series of computational studies [112,113,114]. Interestingly, though most mutations of Asp164 completely inactivate transport, it can be substituted by Glu, with the D164E mutant of EcNhaA keeping the same pH-dependent activity profile as the WT, but with a reduced affinity for Na^+^ and Li^+^ [115,116].

#### 5.2.2. Asp163 and Lys300

The mutation of Asp163 to either Asn, Cys, or Glu was shown to abolish the antiport activity of EcNhaA [106,109,115]. The mutation of the equivalent Asp156 in TtNapA to Asn has the same effect [117]. Due to this and its close proximity to Asp164, Asp163 has long been proposed to be the binding site of the second transported H^+^ of EcNhaA [104]. This was tempting to assume because the electroneutral CPA1 exchangers lack the aspartate at this position, typically having a conserved asparagine [38]. However, recent studies have challenged that assumption [79]. In the structure of EcNhaA [79], Asp163 was shown to form a salt bridge with Lys300, raising the possibility that the second transported proton of EcNhaA originates from this salt bridge. Though this has been supported by molecular dynamics [112], we have shown, using SSM-based electrophysiology, that a double mutant of EcNhaA, D163N/K300Q, is functional in performing electrogenic transport, albeit with a highly reduced turnover rate and reduced affinity for Na^+^ compared to the WT [118]. The analogous mutant in TtNapA, D156N/K305Q seemed inactive in electrophysiological measurements [118], but showed transport activity in fluorescence assays [117]. As mutation of the salt bridge residues was also shown to decrease the thermal stability of EcNhaA; we proposed that the salt bridge has a role in stabilizing the structure of EcNhaA and the related exchangers [118].

Interestingly, the D163N mutant of EcNhaA was recently shown to be unable to bind Na^+^ [83], which the authors concluded to show that Asp163 directly coordinates the Na^+^ ion. How can this be reconciled with the fact that the D163N/K300Q mutant is functional then? One explanation would be that the now-uncompensated positive charge of Lys300 in the D163N mutant repels the Na^+^ ion, preventing binding. In that case, this lack of Na^+^ binding in EcNhaA D163N would neither prove nor disprove Asp163 as a coordinator of the Na^+^ ion.

As mentioned above, in CPA1 exchangers, the Asp163 residue is replaced by asparagine. At the same time, Lys300 is replaced by an arginine [38]. Still, a salt bridge was still identified in the structure of MjNhaP1, between the Glu156 and Arg320 residues [26], with the same finding being made in PaNhaP (between Glu154 and Arg337) [27]. Are those residues equally important for function in CPA1 Na^+^/H^+^ exchangers? The replacement of Arg320 in MjNhaP1 by either Ala or Asp inactivated transport, whereas the mutant R320H was still active [103]. At the same time, the mutant E156A had a transport profile similar to the WT exchanger [103]. Thus, it seems likely that this salt bridge is not as important to the CPA1 Na^+^/H^+^ exchangers compared to those in the CPA2 family, though further studies would be needed in this direction.

#### 5.2.3. Asp133

How can the proposed role of the Asp163–Lys300 salt bridge in proton binding be reconciled with the fact that EcNhaA is still active in the absence of the salt bridge? Recent molecular dynamics simulations [119] explain this by the transporter using an alternate proton transport pathway when the salt bridge is mutated out. Thus, a main contributor to H^+^ binding in Lys300 mutants is proposed to be Asp133, a conserved residue in the unwound section of TMH IV. Binding of the second H^+^ is done either by Asp133 by itself (in the K300R mutant), by Asp133 together with Asp163 (in K300A), or both H^+^ are jointly bound by Asp133 and Asp164 in the D163N/K300Q mutant.

In WT EcNhaA, Asp133 is not indispensable, but its mutation gave rise to some interesting phenotypes. Replacement with Ala kept the same pH-dependent activity profile as the WT, while reducing the affinity for Na^+^ [120]. The D133C mutant, likewise, displayed a reduction in Li^+^ binding affinity, as measured by isothermal titration calorimetry [106]. Interestingly, D133C was shown to possess an altered Li^+^:H^+^ coupled stoichiometry of 4:1 instead of 2:1 for the WT [116]. Finally, the mutation of Asp133 to Ser was reported to preserve the overall pH dependence of EcNhaA, coupled with a reduction of affinities towards Na^+^ and Li^+^ [121]. However, a double mutant, D133S/I134T, loses the ability of catalyzing antiport, unlike the single mutants, D133S and I134T [121]. Interestingly, the double mutant is still capable of binding Li^+^ in a pH-independent fashion, with a higher affinity than the WT [121]. This has been proposed to occur by these mutations altering the electrostatic environment around Asp164, and, thus, affecting its pK_a_ [121].

In CPA1 exchangers, the homologue of Asp133 seems to play a more important role, however. Asp132 of MjNhaP1 has been found to be indispensable for transporter function [103], with the same finding occurring in PaNhaP for the equivalent Asp130 [27]. Indeed, in the structure of PaNhaP obtained in the presence of Tl^+^ (Figure 1c,d), Asp130 interacts with the Tl^+^ ion through a bound water molecule [27]. Thus, the direct contribution to the substrate binding of this aspartate residue appears to be a fundamental difference between CPA1 and CPA2 exchangers. 

#### 5.2.4. Other Residues

In the PaNhaP structure, Glu73, Thr129 (with its main chain carbonyl) and Ser155 also coordinate the Tl^+^ ion (Figure 1d). Glu73 is present on TMH 3 of PaNhaP, which is nominally part of the dimerization domain. There is no homologous residue at this position in EcNhaA [122]. The mutation of Glu73 of PaNhaP to Ala increased the activity of the exchanger, potentially due to the substrate being less tightly bound and, therefore, more easily released [27]. The mutation of Ser155 to Ala had no effect on transport activity, whereas the mutation of Thr129 to Val highly reduced the transport activity [27]. In EcNhaA, the homologue of Thr129 from PaNhaP is Thr132. The mutant T132C has a lower affinity for Na^+^, but keeps an overall similar pH-dependent profile as the WT [120].

### 5.3. “pH Sensor” Region

Most Na^+^/H^+^ exchangers have been found to have a highly pH-dependent profile [71]. This was initially explained by the existence of a “pH sensor” region in the transporter that senses changes in cytoplasmic pH and switches the transporter from a locked, inactive form at acidic pH to an active form at alkaline pH [104]. Many residues present around the entrance of the cytoplasmic funnel have been proposed to be part of the pH sensor of EcNhaA [123,124,125]. These are clustered in the loop between TMHs VIII and IX and on the N-terminal end of TMH IX; they include Glu241, Lys242, Arg245, Lys249, Glu252, Val254, and other residues. The mutation of all these residues affects the pH dependence of the activity profile of EcNhaA

More recently, the pH sensor model of EcNhaA has been challenged, as the transporter has been shown to be active also at acidic pH, under asymmetrical pH conditions, where one side of the transporter is exposed to low pH and the other, where Na^+^ ions are abundant, is at a higher pH [70]. The high pH dependence of the activity profile of EcNhaA and other Na^+^/H^+^ exchangers could, thus, be explained instead by Na^+^ and H^+^ competing for a common binding site [71]. We will discuss this competition mechanism in more detail in Section 6.

### 5.4. Essential Residues in IT Na^+^/H^+^ Exchangers

In comparison to CPA exchangers, much less is known about the amino acid residues that are essential for the function of the IT superfamily Na^+^/H^+^ exchangers. Presumably, future studies would benefit from resolving the structure of at least one IT family Na^+^/H^+^ exchanger.

NhaB exchangers from *V. alginolyticus* (VaNhaB) and *K. pneumoniae* (KpNhaB) have been subjected to mutational studies, revealing two aspartate residues that are very likely to be part of the substrate binding site. In VaNhaB, the mutants of Asp147 were shown to be lacking Na^+^/H^+^ exchange function by fluorescence quenching experiments [126]; although radioactive ^22^Na^+^ efflux experiments showed them to be still capable of ^22^Na^+^/Na^+^ exchange. In a more recent study [92], we showed, using SSM-based electrophysiology, that mutating the equivalent residue, Asp146, of KpNhaB, to Ala reduced the activity of the transporter by about nine-fold, whereas the D146E mutant kept similar characteristics to the WT. The mutation of another aspartate residue in the transmembrane region of the transporter, Asp404, to alanine also had profound effects, drastically reducing the affinity of the protein for Na^+^. However, the most striking phenotype was that of a double mutant, D146A/D404A, which was able to function at three different H^+^:Na^+^ stoichiometries (2:1, 1:1, and 1:2), depending on the pH at which the experiments were performed [92], unlike the WT protein that has a 3:2 stoichiometry. Thus, we could conclude that Asp146 and Asp404 of KpNhaB are part of the substrate binding site, but that there are also additional protonatable residues involved in the substrate binding of KpNhaB that still allow transport function when these two aspartates are absent [92].

Is there a single substrate binding site in KpNhaB or are there two, one for each of the transported Na^+^ ions? Unfortunately, the limited data that we have so far cannot answer this question, and a definitive answer will likely require further functional and structural studies. Certainly, however, the functional data [91] support that the Na^+^ binding site(s) still overlap(s) with the H^+^ binding site(s), as shown by the competition found between the two substrates.

NhaD of *V. cholerae* (VcNhaD) has been subjected to site-directed mutagenesis, allowing the identification of some polar amino acid residues important for its function [127,128]. Thus, the mutation of several residues located at either the membrane–water interface or within the hybrophobic TMHs of VcNhaD predicted by topological analysis led to inactivation of the transporter [127]. Depending on their predicted location in the protein, these were assigned to three clusters [127].

The halotolerant bacterium, *H. hydrothermalis*, Y2 expresses two NhaD proteins, HhNhaD1 and HhNhaD2, that have been investigated in more detail [129,130,131]. These revealed that the N- and C- termini of HhNhaD1 and HhNhaD2 interact and are important for transporter activity [130]. The Glu38 residue of HhNhaD2 was identified to be important in the pH regulation and ion binding [130]. Additionally, seven residues on the predicted TMH XIII were identified to be important for the activity of HhNhaD2 [129]. 

Recently, a comprehensive study analyzing the effects of mutating 25 distinct residues of HhNhaD1 was published [96]. These mutations were performed using the predicted structure of HhNhaD1 as the basis, which, as previously mentioned, resembles that of the dicarboxylate transporter, VcINDY [132]. In VcINDY, two Na^+^ binding sites, Na1 and Na2, were identified. The mutation to alanine of the homologous residues in HhNhaD1 (Ser162, Asp166, and Asn167) to those forming the Na1 site of VcINDY resulted in a complete loss of function of the mutant [96]. A similar result was obtained when mutating Gly204 of HhNhaD1. Other mutations in HhNhaD1 were performed on residues belonging to the proposed second Na^+^ binding site: Ser401, Asp405, Asn406, Gly436, and Gly442. The mutation of all these residues to alanine resulted in a complete loss of function, with the exception of the S401A and N406A mutants, which could still partly support Na^+^ export at lower concentrations [96]. Analogous to the two aspartates (Asp163 and Asp164) of EcNhaA, Asp166 and Asp405 were proposed to be involved in H^+^ transport in HhNhaD1 [96]. Their presence in the predicted hairpin regions of HhNhaD1 evokes the predicted location in the hairpins of Asp146 and Asp404 in KpNhaB (Figure 2b). Finally, the authors proposed the existence of a pH sensor region at the cytoplasmic side of HhNhaD1, with residues Glu64, Glu65, Arg454, and Arg464 proposed to be important for the pH regulation of the transporter [96].

## 6. Transport Mechanism and Stoichiometry

### 6.1. pH Regulation of Na^+^/H^+^ Exchangers

**Figure 3:**
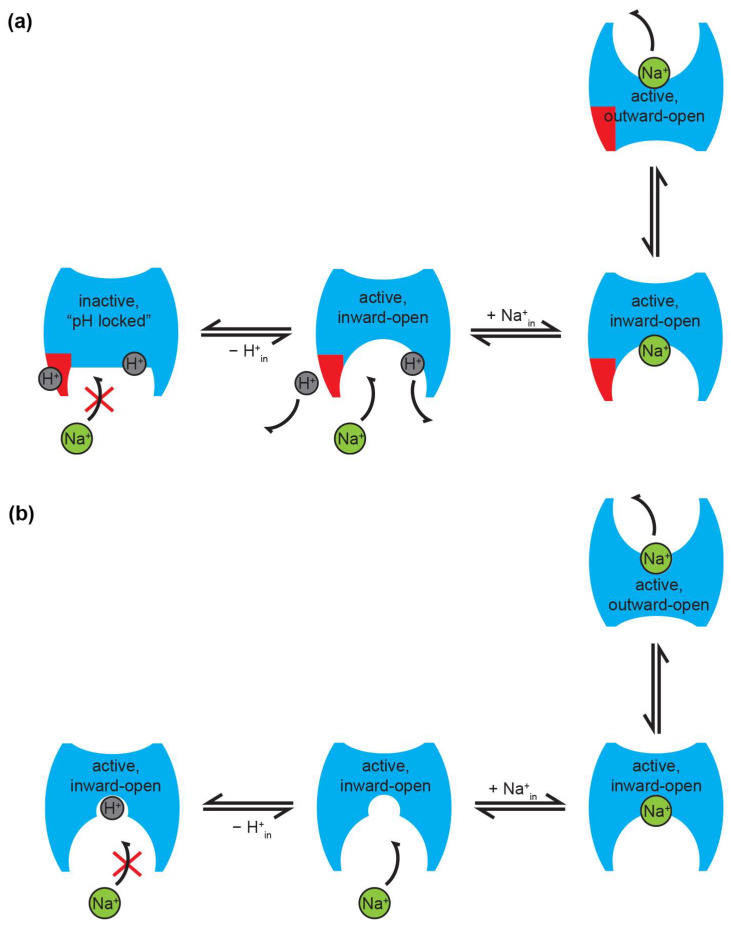
Proposed modes of pH regulation of Na^+^/H^+^ exchanger activity. (**a**) In the pH sensor model, an acidic cytoplasmic pH locks the protein in an inactive form. The pH sensor region (shown in red) is protonated at acidic pH, preventing Na^+^ from accessing the binding site. At alkaline pH, the pH sensor is deprotonated, allowing Na^+^ binding, followed by translocation across the membrane. Note that an additional H^+^ is also shown bound to the substrate binding site(s) of the transporter. In order to ensure correct coupling, this has to be released before Na^+^ can be translocated. (**b**) In the competition model, both substrates compete for the same binding site; thus, activity is down-regulated at low pH, due to H^+^ outcompeting Na^+^ for binding to the exchanger. Note that, in this model, all forms are active at all times, and are, thus, capable of performing transport in the presence of substrate. In both panels, the notations “in” and “out” refer to cytoplasmic and periplasmic, respectively. The figure shows schematic transport for a 1:1 H^+^:Na^+^ stoichiometry, but is equally valid for other stoichiometries, such as 2:1 for EcNhaA. The red cross in both panels shows that under the conditions depicted Na^+^ cannot bind to the exchanger. For simplicity, in both panels, only half of the transport cycle (Na^+^ binding and translocation across the membrane) is shown. A schematic of the full transport cycle in the competition mechanism can be found in [71].

Perhaps the most prominent feature of prokaryotic Na^+^/H^+^ exchangers is the steep pH dependence of their transport activity. In EcNhaA reconstituted in liposomes, the exchange activity, measured as the rate of Na^+^ efflux, is increased by three orders of magnitude when the pH changes from 6.5 to 8 [76]. To explain this, taking into account the possible existence of a pH sensor region (see Section 5.3), an allosteric mechanism of pH regulation was proposed. This model states that the alteration of the protonation state in the pH sensor region is transduced into a conformational change that triggers the activation of the exchange in EcNhaA [24,115]. The down-regulation at acidic pH is explained as a blocking of the periplasmic passage by an ion barrier formed partly by helix XIp, and, therefore, only a portion of the cation binding site is accessible to the cation passage (Figure 3a). In response to a signal from the pH sensor, a conformational change in helices IX and X triggers a reorientation of the active site. This process exposes the Na^+^ binding site to the cytoplasmic passage, and removes the periplasmic ion barrier, leaving the active site available for the cation binding [104], thus “activating” the exchanger, which can then function according to the classical alternating access model [133]. In this way, the binding of Na^+^ or Li^+^ results in an electrical imbalance in the TMH XI-IV assembly that leads to the orientation of the active site to the periplasm, and the sealing off from the cytoplasm. Upon cation release, residues in the binding site are protonated, which induces a conformational change that re-exposes the active site to the periplasm, completing the alternating access cycle. This mechanism is supported by mutagenesis effects on regions far away from the binding site (see Section 5.3), by the experimental detection of pH-induced and ligand-induced conformational changes [134], as well as computational studies [113,135]. 

It should be noted that regulation via a pH sensor is equally possible if there is one or more binding sites; therefore, the model is valid in either case. In the case of a single binding site, however, the pH sensor would essentially be redundant, as competition would ensure down-regulation at acidic pH anyway (see below). Moreover, irrespective of whether there is a single or multiple substrate binding sites in the transporter, in order to ensure coupled transport and correct function according to the alternating access model [133], translocation of one substrate across the membrane can only be performed when the other substrate is not bound to the exchanger.

An alternative model has been proposed to explain the pH regulation of Na^+^/H^+^ exchangers, relying on competition between the substrates for a common binding site (Figure 3b). This model has been shown to be valid in the CPA2 exchangers, EcNhaA [70], NhaA from *H. pylori* [108], NhaA from *S. typhimurium* [136], TtNapA [92], the CPA1 exchangers MjNhaP1 [105], and PaNhaP [73], but also in the IT exchanger, KpNhaB [91]. 

Unlike the allosteric model, the competition-based model explains that the pH regulation in EcNhaA and other Na^+^/H^+^ exchangers is performed via its active site. SSM-based electrophysiological measurements performed on inside-out membrane vesicles expressing EcNhaA and right-side out EcNhaA proteoliposomes evidenced a bell-shaped pH dependence of its electrogenic activity, indicating that the transporter is down-regulated at acidic, as well as at alkaline pH values under conditions when the pH is kept the same across the membrane (pH_in_ = pH_out_) [70]. The down-regulation at acidic pH results from the competition of Na^+^ and H^+^ for a single common binding site, which is evidenced as an increment in the K_m_ for Na^+^. Supporting this explanation, the v_max_ obtained at saturating Na^+^ concentrations at acidic pHs does not change substantially with the pH. By contrast, the down-regulation at alkaline pH is attributed to the decrease of v_max_, due to H^+^ depletion [70].

Under symmetrical conditions of pH (pH_in_ = pH_out_), transport is abolished for pH < 6.5 as a consequence of substrate competition. However, a remarkable finding was the persistently high transport activity demonstrated under asymmetrical conditions (pH 5 in the intravesicular compartment, and high pH outside), which argues against the pH regulation via a pH sensor [70]. 

To explain these findings, a minimal kinetic model based on a single common binding site for H^+^ and Na^+^ was designed and validated against the experimental data [70]. In this model, it was assumed that the binding site is identical when exposed to the cytoplasmic and extracellular sides, and the inward and outward open conformations of the carrier have the same energy [71]. This was justified by the experimental evidence that the kinetic parameters measured in the physiological and reverse mode of transport are similar for EcNhaA. Although the model supports a symmetrical function of the transporter, the asymmetry suggested by the alternate access mechanism as an effective way to perform the function of a transporter [133] is achieved by the interaction with the asymmetrically distributed counter-substrate. By the competitive interaction of Na^+^ and H^+^ at the binding site, alkaline pH at the Na^+^ uptake side of the transporter creates a low K_m_ of ≈ 10 mM, whereas at the release side at acidic pH, K_m_ ≈ 100 mM. This enables effective counter-transport of Na^+^ and H^+^ [70].

What makes the competition model compelling is that it acts as an inbuilt regulator of protein activity that does not need any other regions of the protein other than the substrate binding site, though it does not preclude the existence of any such regions either. Thus, activity is down-regulated naturally when one of the substrates becomes too low in concentration relative to the other [71], ensuring that transporter activity slows down or stops under conditions where it would start to be detrimental for the cell.

Interestingly, the competition-based mechanism has been expanded to Na^+^/H^+^ antiporters from the IT superfamily; specifically, from the NhaB family. It was shown that similar to CPA2 members, the down-regulation at acidic pH observed for KpNhaB is mainly a consequence of a Na^+^ and H^+^ competing for a common binding site [91]. This points to a mechanism shared by prokaryotic Na^+^/H^+^ exchangers in which the competition for a single common binding site constitutes an effective regulation strategy [71]. 

Recently, a study on EcNhaA has challenged the finding that Na^+^ cannot bind when the pH at the binding site is low [83]. Using the scintillation proximity assay (SPA) technique, it was shown that at pH values between 4 and 6, EcNhaA can still bind around 40% of the amount of Na^+^ that it can bind at pH 8. No Na^+^ binding was found for the D163N mutant under any conditions [83]. Does this disprove the competition model? Very likely not. What the SPA measurements cannot show is whether Na^+^ actually binds to an active binding site at pH 4. Certainly, previous experiments have shown no active transport under these conditions. It is possible then that the Na^+^ bound at low pH is not fully coordinated by the binding site because, for example, Asp164 is protonated. This might, thus, prevent the transporter from undergoing the required conformational transitions that switch between the inward- and outward-open states. Along the same lines, a recent study showed that MjNhaP1 can bind K^+^ (which is not a substrate), but is unable to translocate it and, thus, perform K^+^/H^+^ antiport [72].

### 6.2. Stoichiometry

What are the molecular determinants of electrogenicity and stoichiometry in prokaryotic Na^+^/H^+^ antiporters? This is mainly still an open question. In line with the single common binding site for both substrates, it was suggested in EcNhaA that the residues from the active site, Asp163 and Asp164, would constitute the binding site for H^+^ and Na^+^ in a “2-aspartates” model [70]. This mechanistic model explains that the conformational transitions of the fully occupied binding site are associated with a displacement of the two negatively charged aspartate residues (Asp163 and Asp164), and one Na^+^ or two H^+^ ions, respectively. In the case of Na^+^ translocation, this leads to a net charge displacement of one negative charge, which makes it the electrogenic partial step, and supports the global electrogenicity. In contrast to this, structural data indicated that Asp163 participates in a salt-bridge-type interaction with Lys300, which led to the proposal that the salt bridge would be the actual binding site for a second H^+^ [79]. Unlike the “2-aspartates” model, the “Asp163-Lys300” model states that whereas the two aspartates in positions 163 and 164 bind the Na^+^, only Asp164 binds one of the H^+^ ions, and the salt bridge would support the binding of the other H^+^. 

Although experimental evidence collected from the mutagenesis of residues in the binding site pointed to a role of Lys300 more in protein stability rather than a direct responsibility for the H^+^ binding [137], it can be conceived that the two mechanistic models are not mutually exclusive. In fact, the detection of electrogenic exchange when Asp163 and Lys300 are substituted for non-charged amino acids supports some versatility in the active site, such that, in the absence of essential residues, other amino acids in the vicinity can take over and support transport activity, as discussed in Section 5.2.2. In this scenario, the salt bridge might be considered as a transitory state that confers rigidity to the protein, and assures no leak transport in absence of substrate, such that Na^+^ and H^+^ translocation are strictly coupled events.

In the electroneutral CPA1 members, the homologue of Asp163 is usually replaced by an Asn residue. Does that mean that this is why these transporters are electroneutral? The study presenting the CPA motif [38] proposes that residues 6, 7, and 8 of the motif are crucial for electrogenicity; although, as we discussed in Section 5, the replacement of all these residues, except for Asp164, can still yield a functional transporter. It is very likely that the answer is more complex than that, as evidenced by the fact that the NHA2 human transporter that carries the “double-aspartate” model is electroneutral [29].

NhaB exchangers are electrogenic with a 3:2 H^+^:Na^+^ stoichiometry [45]. As previously mentioned in Section 5.4, we found that the mutation of two key aspartate residues could alter the stoichiometry and even allow the transporter to function under different stoichiometries depending on pH [92]. This indicates that the line between a normally functioning transporter and a completely abnormal variant might be just a few mutations away. However, a precise explanation of the observed phenotype will likely require a high-resolution structure of the exchanger. Due to the high sequence dissimilarities between IT and CPA Na^+^/H^+^ exchangers, it is likely that the overall architecture of the binding site of NhaB is significantly different to that of NhaA and other CPA members, even though they seem to be functioning according to the similar mechanism of competition.

## 7. Outlook

As evidenced from this review, a large amount of effort has been poured into deciphering the inner workings of prokaryotic Na^+^/H^+^ exchangers. Open questions still remain, and we will list only a few. What are the exact residues coordinating the substrate ions? What are the molecular determinants of electrogenicity that separate electrogenic from electroneutral exchangers? What are the main structural and mechanistic features that distinguish between CPA-type and other Na^+^/H^+^ exchangers? Perhaps the most important question of all is: how can we translate this current and future knowledge into medically relevant information, either for understanding and treating disease that affects Na^+^/H^+^ expression and function, or for the development of novel antibacterial drugs? Certainly, future work will point us more and more towards this direction.

## Figures and Tables

**Table 1:** Physiological role of some prokaryotic Na^+^/H^+^ exchangers from different families. NA denotes an unknown stoichiometry.

Superfamily	Transporter	H^+^:Na^+^ Stoichiometry	Physiological Role	Organism	References
**CPA**	NhaP	1:1	pH regulation, H^+^ export	*M. jannaschii*	[74]
		1:1	Resistance to K^+^ at alkaline pH, K^+^/H^+^ exchange	*V. cholerae*	[75]
	NhaA	2:1	Salt resistance, Na^+^ (Li^+^) export	*E. coli*	[76]
	NapA	2:1	Salt resistance	*T. thermophilus*	[77]
**IT**	NhaB	3:2	pH homeostasis at neutral pH conditions and low concentrations of Na^+^	*E. coli*	[44]
	NhaC	NA (electrogenic)	Salt tolerance and pH homeostasis at alkaline conditions	*B. firmus*	[46]
	NhaD	NA	Salt tolerance and pH homeostasis at alkaline conditions	*V. cholerae*	[49]
	NhaE	NA	Salt tolerance at neutral pH	*R. marinus*	[51]
**Mrp**	Mrp	NA (electrogenic)	Salt resistance, pH homeostasis	*B. subtilis*	[78]
		NA (electrogenic)	Salt resistance, bile acid export	*V. cholerae*	[67]

## References

[1] Krulwich T.A., Sachs G., Padan E. (2011). Molecular aspects of bacterial pH sensing and homeostasis. Nat. Rev. Microbiol..

[2] Marshall W.J., Marshall W.J., Lapsley M., Day A.P., Ayling R.M. (2014). CHAPTER 5—Hydrogen ion homoeostasis and tissue oxygenation and their disorders. Clinical Biochemistry: Metabolic and Clinical Aspects.

[3] Padan E., Bibi E., Ito M., Krulwich T.A. (2005). Alkaline pH homeostasis in bacteria: New insights. Biochim. Biophys. Acta.

[4] Slonczewski J.L., Fujisawa M., Dopson M., Krulwich T.A. (2009). Cytoplasmic pH measurement and homeostasis in bacteria and archaea. Adv. Microb. Physiol..

[5] Murphy E., Eisner D.A. (2009). Regulation of intracellular and mitochondrial sodium in health and disease. Circ. Res..

[6] Zacchia M., Abategiovanni M.L., Stratigis S., Capasso G. (2016). Potassium: From Physiology to Clinical Implications. Kidney Dis..

[7] Gao A.Y.L., Lourdin-De Filippis E., Orlowski J., McKinney R.A. (2022). Roles of Endomembrane Alkali Cation/Proton Exchangers in Synaptic Function and Neurodevelopmental Disorders. Front. Physiol..

[8] Padan E., Landau M. (2016). Sodium-Proton (Na(+)/H(+)) Antiporters: Properties and Roles in Health and Disease. Met. Ions Life Sci..

[9] Pedersen S.F., Counillon L. (2019). The SLC9A-C Mammalian Na(+)/H(+) Exchanger Family: Molecules, Mechanisms, and Physiology. Physiol. Rev..

[10] Fuster D.G., Alexander R.T. (2014). Traditional and emerging roles for the SLC9 Na^+^/H^+^ exchangers. Pflugers Arch..

[11] Donowitz M., Ming Tse C., Fuster D. (2013). SLC9/NHE gene family, a plasma membrane and organellar family of Na(+)/H(+) exchangers. Mol. Aspects Med..

[12] Chen S.R., Chen M., Deng S.L., Hao X.X., Wang X.X., Liu Y.X. (2016). Sodium-hydrogen exchanger NHA1 and NHA2 control sperm motility and male fertility. Cell Death Dis..

[13] Kondapalli K.C., Todd Alexander R., Pluznick J.L., Rao R. (2017). NHA2 is expressed in distal nephron and regulated by dietary sodium. J. Physiol. Biochem..

[14] Deisl C., Simonin A., Anderegg M., Albano G., Kovacs G., Ackermann D., Moch H., Dolci W., Thorens B., Hediger M.A. (2013). Sodium/hydrogen exchanger NHA2 is critical for insulin secretion in beta-cells. Proc. Natl. Acad. Sci. USA.

[15] Cavarocchi E., Whitfield M., Chargui A., Stouvenel L., Lores P., Coutton C., Arnoult C., Santulli P., Patrat C., Thierry-Mieg N. (2021). The sodium/proton exchanger SLC9C1 (sNHE) is essential for human sperm motility and fertility. Clin. Genet..

[16] Orlowski J., Grinstein S. (2011). Na^+^/H^+^ exchangers. Compr. Physiol..

[17] Xu H., Ghishan F.K., Kiela P.R. (2018). SLC9 Gene Family: Function, Expression, and Regulation. Compr. Physiol..

[18] Anderegg M.A., Gyimesi G., Ho T.M., Hediger M.A., Fuster D.G. (2022). The Less Well-Known Little Brothers: The SLC9B/NHA Sodium Proton Exchanger Subfamily-Structure, Function, Regulation and Potential Drug-Target Approaches. Front. Physiol..

[19] Landau M., Herz K., Padan E., Ben-Tal N. (2007). Model structure of the Na^+^/H^+^ exchanger 1 (NHE1): Functional and clinical implications. J. Biol. Chem..

[20] Padan E. (2014). Functional and structural dynamics of NhaA, a prototype for Na(+) and H(+) antiporters, which are responsible for Na(+) and H(+) homeostasis in cells. Biochim. Biophys. Acta.

[21] Minato Y., Ghosh A., Faulkner W.J., Lind E.J., Schesser Bartra S., Plano G.V., Jarrett C.O., Hinnebusch B.J., Winogrodzki J., Dibrov P. (2013). Na+/H+ antiport is essential for Yersinia pestis virulence. Infect. Immun..

[22] Karpel R., Alon T., Glaser G., Schuldiner S., Padan E. (1991). Expression of a sodium proton antiporter (NhaA) in *Escherichia coli* is induced by Na^+^ and Li^+^ ions. J. Biol. Chem..

[23] Brett C.L., Donowitz M., Rao R. (2005). Evolutionary origins of eukaryotic sodium/proton exchangers. Am. J. Physiol. Cell Physiol..

[24] Hunte C., Screpanti E., Venturi M., Rimon A., Padan E., Michel H. (2005). Structure of a Na^+^/H^+^ antiporter and insights into mechanism of action and regulation by pH. Nature.

[25] Lee C., Kang H.J., von Ballmoos C., Newstead S., Uzdavinys P., Dotson D.L., Iwata S., Beckstein O., Cameron A.D., Drew D. (2013). A two-domain elevator mechanism for sodium/proton antiport. Nature.

[26] Paulino C., Wohlert D., Kapotova E., Yildiz O., Kuhlbrandt W. (2014). Structure and transport mechanism of the sodium/proton antiporter MjNhaP1. eLife.

[27] Wohlert D., Kuhlbrandt W., Yildiz O. (2014). Structure and substrate ion binding in the sodium/proton antiporter PaNhaP. eLife.

[28] Dong Y., Gao Y., Ilie A., Kim D., Boucher A., Li B., Zhang X.C., Orlowski J., Zhao Y. (2021). Structure and mechanism of the human NHE1-CHP1 complex. Nat. Commun..

[29] Matsuoka R., Fudim R., Jung S., Zhang C., Bazzone A., Chatzikyriakidou Y., Robinson C.V., Nomura N., Iwata S., Landreh M. (2022). Structure, mechanism and lipid-mediated remodeling of the mammalian Na(+)/H(+) exchanger NHA2. Nat. Struct. Mol. Biol..

[30] Dwivedi M. (2021). Site-directed mutations reflecting functional and structural properties of Ec-NhaA. Biochimie.

[31] Saier M.H., Reddy V.S., Tamang D.G., Vastermark A. (2014). The transporter classification database. Nucleic Acids Res..

[32] Bruford E.A., Lush M.J., Wright M.W., Sneddon T.P., Povey S., Birney E. (2008). The HGNC Database in 2008: A resource for the human genome. Nucleic Acids Res..

[33] Saier M.H., Tran C.V., Barabote R.D. (2006). TCDB: The Transporter Classification Database for membrane transport protein analyses and information. Nucleic Acids Res..

[34] Saier M.H., Reddy V.S., Tsu B.V., Ahmed M.S., Li C., Moreno-Hagelsieb G. (2016). The Transporter Classification Database (TCDB): Recent advances. Nucleic Acids Res..

[35] Saier M.H., Reddy V.S., Moreno-Hagelsieb G., Hendargo K.J., Zhang Y., Iddamsetty V., Lam K.J.K., Tian N., Russum S., Wang J. (2021). The Transporter Classification Database (TCDB): 2021 update. Nucleic Acids Res..

[36] Shao L., Xu T., Zheng X., Shao D., Zhang H., Chen H., Zhang Z., Yan M., Abdel-Motaal H., Jiang J. (2021). A novel three-TMH Na(+)/H(+) antiporter and the functional role of its oligomerization. J. Mol. Biol..

[37] Lu S., Wang J., Chitsaz F., Derbyshire M.K., Geer R.C., Gonzales N.R., Gwadz M., Hurwitz D.I., Marchler G.H., Song J.S. (2020). CDD/SPARCLE: The conserved domain database in 2020. Nucleic Acids Res..

[38] Masrati G., Dwivedi M., Rimon A., Gluck-Margolin Y., Kessel A., Ashkenazy H., Mayrose I., Padan E., Ben-Tal N. (2018). Broad phylogenetic analysis of cation/proton antiporters reveals transport determinants. Nat. Commun..

[39] Mourin M., Wai A., O’Neil J., Hausner G., Dibrov P. (2019). Physiological, Structural, and Functional Analysis of the Paralogous Cation-Proton Antiporters of NhaP Type from Vibrio cholerae. Int. J. Mol. Sci..

[40] Xiang M., Feng M., Muend S., Rao R. (2007). A human Na^+^/H^+^ antiporter sharing evolutionary origins with bacterial NhaA may be a candidate gene for essential hypertension. Proc. Natl. Acad. Sci. USA.

[41] Padan E., Michel H. (2015). NhaA: A Unique Structural Fold of Secondary Active Transporters. Isr. J. Chem..

[42] Prakash S., Cooper G., Singhi S., Saier M.H. (2003). The ion transporter superfamily. Biochim. Biophys. Acta.

[43] Pinner E., Padan E., Schuldiner S. (1992). Cloning, sequencing, and expression of the nhaB gene, encoding a Na^+^/H^+^ antiporter in *Escherichia coli*. J. Biol. Chem..

[44] Pinner E., Kotler Y., Padan E., Schuldiner S. (1993). Physiological role of nhaB, a specific Na^+^/H^+^ antiporter in *Escherichia coli*. J. Biol. Chem..

[45] Pinner E., Padan E., Schuldiner S. (1994). Kinetic properties of NhaB, a Na+/H+ antiporter from *Escherichia coli*. J. Biol. Chem..

[46] Ito M., Guffanti A.A., Zemsky J., Ivey D.M., Krulwich T.A. (1997). Role of the nhaC-encoded Na^+^/H^+^ antiporter of alkaliphilic Bacillus firmus OF_4_. J. Bacteriol..

[47] Haja D.K., Adams M.W.W. (2021). pH Homeostasis and Sodium Ion Pumping by Multiple Resistance and pH Antiporters in Pyrococcus furiosus. Front. Microbiol..

[48] Nozaki K., Kuroda T., Mizushima T., Tsuchiya T. (1998). A new Na^+^/H^+^ antiporter, NhaD, of Vibrio parahaemolyticus. Biochim. Biophys. Acta.

[49] Dzioba J., Ostroumov E., Winogrodzki A., Dibrov P. (2002). Cloning, functional expression in *Escherichia coli* and primary characterization of a new Na^+^/H^+^ antiporter, NhaD, of Vibrio cholerae. Mol. Cell. Biochem..

[50] Wang Y., Song N., Yang L., Abdel-Motaal H., Zhang R., Zhang Z., Meng F., Jiang J. (2017). A novel NhaD-type Na(+)/H(+) antiporter from the moderate halophile and alkaliphile Halomonas alkaliphila. Can. J. Microbiol..

[51] Melo A.M., Felix N.A., Carita J.N., Saraiva L.M., Teixeira M. (2006). The Na^+^/H^+^ antiporter of the thermohalophilic bacterium Rhodothermus marinus. Biochem. Biophys. Res. Commun..

[52] Sousa P.M., Videira M.A., Vorburger T., Silva S.T., Moir J.W., Steuber J., Melo A.M. (2013). The novel NhaE-type Na(+)/H (+) antiporter of the pathogenic bacterium Neisseria meningitidis. Arch. Microbiol..

[53] Ito M., Morino M., Krulwich T.A. (2017). Mrp Antiporters Have Important Roles in Diverse Bacteria and Archaea. Front. Microbiol..

[54] Kudo T., Hino M., Kitada M., Horikoshi K. (1990). DNA sequences required for the alkalophily of *Bacillus* sp. strain C-125 are located close together on its chromosomal DNA. J. Bacteriol..

[55] Hamamoto T., Hashimoto M., Hino M., Kitada M., Seto Y., Kudo T., Horikoshi K. (1994). Characterization of a gene responsible for the Na^+^/H^+^ antiporter system of alkalophilic Bacillus species strain C-125. Mol. Microbiol..

[56] Ohyama T., Imaizumi R., Igarashi K., Kobayashi H. (1992). *Escherichia coli* is able to grow with negligible sodium ion extrusion activity at alkaline pH. J. Bacteriol..

[57] Padan E., Maisler N., Taglicht D., Karpel R., Schuldiner S. (1989). Deletion of ant in *Escherichia coli* reveals its function in adaptation to high salinity and an alternative Na^+^/H^+^ antiporter system(s). J. Biol. Chem..

[58] Taglicht D., Padan E., Schuldiner S. (1993). Proton-sodium stoichiometry of NhaA, an electrogenic antiporter from *Escherichia coli*. J. Biol. Chem..

[59] Padan E., Venturi M., Gerchman Y., Dover N. (2001). Na(+)/H(+) antiporters. Biochim. Biophys. Acta.

[60] Herz K., Vimont S., Padan E., Berche P. (2003). Roles of NhaA, NhaB, and NhaD Na^+^/H^+^ antiporters in survival of Vibrio cholerae in a saline environment. J. Bacteriol..

[61] Kuroda T., Mizushima T., Tsuchiya T. (2005). Physiological roles of three Na^+^/H^+^ antiporters in the halophilic bacterium Vibrio parahaemolyticus. Microbiol. Immunol..

[62] Foreman S., Ferrara K., Hreha T.N., Duran-Pinedo A.E., Frias-Lopez J., Barquera B. (2021). Genetic and Biochemical Characterization of the Na(+)/H(+) Antiporters of Pseudomonas aeruginosa. J. Bacteriol..

[63] Schubiger C.B., Hoang K.H.T., Hase C.C. (2020). Sodium antiporters of Pseudomonas aeruginosa in challenging conditions: Effects on growth, biofilm formation, and swarming motility. J. Genet. Eng. Biotechnol..

[64] Kuroda T., Fujita N., Utsugi J., Kuroda M., Mizushima T., Tsuchiya T. (2004). A major Li(+) extrusion system NhaB of Pseudomonas aeruginosa: Comparison with the major Na(+) extrusion system NhaP. Microbiol. Immunol..

[65] Liew C.W., Illias R.M., Mahadi N.M., Najimudin N. (2007). Expression of the Na^+^/H^+^ antiporter gene (g1-nhaC) of alkaliphilic *Bacillus* sp. G1 in *Escherichia coli*. FEMS Microbiol. Lett..

[66] Wei Y., Guffanti A.A., Ito M., Krulwich T.A. (2000). *Bacillus subtilis* YqkI is a novel malic/Na+-lactate antiporter that enhances growth on malate at low protonmotive force. J. Biol. Chem..

[67] Dzioba-Winogrodzki J., Winogrodzki O., Krulwich T.A., Boin M.A., Hase C.C., Dibrov P. (2009). The Vibrio cholerae Mrp system: Cation/proton antiport properties and enhancement of bile salt resistance in a heterologous host. J. Mol. Microbiol. Biotechnol..

[68] Kosono S., Ohashi Y., Kawamura F., Kitada M., Kudo T. (2000). Function of a principal Na(+)/H(+) antiporter, ShaA, is required for initiation of sporulation in *Bacillus subtilis*. J. Bacteriol..

[69] Kosono S., Haga K., Tomizawa R., Kajiyama Y., Hatano K., Takeda S., Wakai Y., Hino M., Kudo T. (2005). Characterization of a multigene-encoded sodium/hydrogen antiporter (sha) from Pseudomonas aeruginosa: Its involvement in pathogenesis. J. Bacteriol..

[70] Mager T., Rimon A., Padan E., Fendler K. (2011). Transport mechanism and pH regulation of the Na^+^/H^+^ antiporter NhaA from *Escherichia coli*: An electrophysiological study. J. Biol. Chem..

[71] Calinescu O., Fendler K. (2015). A universal mechanism for transport and regulation of CPA sodium proton exchangers. Biol. Chem..

[72] Warnau J., Wohlert D., Okazaki K.I., Yildiz O., Gamiz-Hernandez A.P., Kaila V.R.I., Kuhlbrandt W., Hummer G. (2020). Ion Binding and Selectivity of the Na^+^/H^+^ Antiporter MjNhaP1 from Experiment and Simulation. J. Phys. Chem. B.

[73] Calinescu O., Linder M., Wohlert D., Yildiz O., Kuhlbrandt W., Fendler K. (2016). Electrogenic Cation Binding in the Electroneutral Na^+^/H^+^ Antiporter of Pyrococcus abyssi. J. Biol. Chem..

[74] Vinothkumar K.R., Smits S.H., Kuhlbrandt W. (2005). pH-induced structural change in a sodium/proton antiporter from Methanococcus jannaschii. EMBO J..

[75] Mourin M., Schubiger C.B., Resch C.T., Hase C.C., Dibrov P. (2017). Physiology of the Vc-NhaP paralogous group of cation-proton antiporters in Vibrio cholerae. Mol. Cell. Biochem..

[76] Taglicht D., Padan E., Schuldiner S. (1991). Overproduction and purification of a functional Na^+^/H^+^ antiporter coded by nhaA (ant) from *Escherichia coli*. J. Biol. Chem..

[77] Furrer E.M., Ronchetti M.F., Verrey F., Pos K.M. (2007). Functional characterization of a NapA Na^+^/H^+^ antiporter from Thermus thermophilus. FEBS Lett..

[78] Ito M., Guffanti A.A., Oudega B., Krulwich T.A. (1999). mrp, a multigene, multifunctional locus in *Bacillus subtilis* with roles in resistance to cholate and to Na^+^ and in pH homeostasis. J. Bacteriol..

[79] Lee C., Yashiro S., Dotson D.L., Uzdavinys P., Iwata S., Sansom M.S., von Ballmoos C., Beckstein O., Drew D., Cameron A.D. (2014). Crystal structure of the sodium-proton antiporter NhaA dimer and new mechanistic insights. J. Gen. Physiol..

[80] Berman H.M., Westbrook J., Feng Z., Gilliland G., Bhat T.N., Weissig H., Shindyalov I.N., Bourne P.E. (2000). The Protein Data Bank. Nucleic Acids Res..

[81] Hu N.J., Iwata S., Cameron A.D., Drew D. (2011). Crystal structure of a bacterial homologue of the bile acid sodium symporter ASBT. Nature.

[82] Zhou X., Levin E.J., Pan Y., McCoy J.G., Sharma R., Kloss B., Bruni R., Quick M., Zhou M. (2014). Structural basis of the alternating-access mechanism in a bile acid transporter. Nature.

[83] Quick M., Dwivedi M., Padan E. (2021). Insight into the direct interaction of Na^+^ with NhaA and mechanistic implications. Sci. Rep..

[84] Rimon A., Tzubery T., Padan E. (2007). Monomers of the NhaA Na+/H+ antiporter of *Escherichia coli* are fully functional yet dimers are beneficial under extreme stress conditions at alkaline pH in the presence of Na^+^ or Li^+^. J. Biol. Chem..

[85] Padan E., Danieli T., Keren Y., Alkoby D., Masrati G., Haliloglu T., Ben-Tal N., Rimon A. (2015). NhaA antiporter functions using 10 helices, and an additional 2 contribute to assembly/stability. Proc. Natl. Acad. Sci. USA.

[86] Herz K., Rimon A., Jeschke G., Padan E. (2009). Beta-sheet-dependent dimerization is essential for the stability of NhaA Na^+^/H^+^ antiporter. J. Biol. Chem..

[87] Okazaki K.I., Wohlert D., Warnau J., Jung H., Yildiz O., Kuhlbrandt W., Hummer G. (2019). Mechanism of the electroneutral sodium/proton antiporter PaNhaP from transition-path shooting. Nat. Commun..

[88] Masrati G., Mondal R., Rimon A., Kessel A., Padan E., Lindahl E., Ben-Tal N. (2020). An angular motion of a conserved four-helix bundle facilitates alternating access transport in the TtNapA and EcNhaA transporters. Proc. Natl. Acad. Sci. USA.

[89] Jumper J., Evans R., Pritzel A., Green T., Figurnov M., Ronneberger O., Tunyasuvunakool K., Bates R., Zidek A., Potapenko A. (2021). Highly accurate protein structure prediction with AlphaFold. Nature.

[90] Varadi M., Anyango S., Deshpande M., Nair S., Natassia C., Yordanova G., Yuan D., Stroe O., Wood G., Laydon A. (2022). AlphaFold Protein Structure Database: Massively expanding the structural coverage of protein-sequence space with high-accuracy models. Nucleic Acids Res..

[91] Patino-Ruiz M., Ganea C., Fendler K., Calinescu O. (2017). Competition is the basis of the transport mechanism of the NhaB Na^+^/H^+^ exchanger from Klebsiella pneumoniae. PLoS ONE.

[92] Patino-Ruiz M., Fendler K., Calinescu O. (2019). Mutation of two key aspartate residues alters stoichiometry of the NhaB Na^+^/H^+^ exchanger from Klebsiella pneumoniae. Sci. Rep..

[93] Pereira J., Simpkin A.J., Hartmann M.D., Rigden D.J., Keegan R.M., Lupas A.N. (2021). High-accuracy protein structure prediction in CASP14. Proteins.

[94] UniProt C. (2021). UniProt: The universal protein knowledgebase in 2021. Nucleic Acids Res..

[95] Mancusso R., Gregorio G.G., Liu Q., Wang D.N. (2012). Structure and mechanism of a bacterial sodium-dependent dicarboxylate transporter. Nature.

[96] Lv P., Li Y., Wang R., Zhang Y., Wang W., Liu Y., Shang Y., Su D., Wang W., Yang C. (2022). The pH sensor and ion binding of NhaD Na^+^/H^+^ antiporter from IT superfamily. Mol. Microbiol..

[97] Morino M., Suzuki T., Ito M., Krulwich T.A. (2014). Purification and functional reconstitution of a seven-subunit mrp-type na+/h+ antiporter. J. Bacteriol..

[98] Steiner J., Sazanov L. (2020). Structure and mechanism of the Mrp complex, an ancient cation/proton antiporter. eLife.

[99] Li B., Zhang K., Nie Y., Wang X., Zhao Y., Zhang X.C., Wu X.L. (2020). Structure of the Dietzia Mrp complex reveals molecular mechanism of this giant bacterial sodium proton pump. Proc. Natl. Acad. Sci. USA.

[100] Morino M., Natsui S., Swartz T.H., Krulwich T.A., Ito M. (2008). Single gene deletions of mrpA to mrpG and mrpE point mutations affect activity of the Mrp Na^+^/H^+^ antiporter of alkaliphilic Bacillus and formation of hetero-oligomeric Mrp complexes. J. Bacteriol..

[101] Swartz T.H., Ito M., Hicks D.B., Nuqui M., Guffanti A.A., Krulwich T.A. (2005). The Mrp Na^+^/H^+^ antiporter increases the activity of the malate:quinone oxidoreductase of an *Escherichia coli* respiratory mutant. J. Bacteriol..

[102] Ito M., Guffanti A.A., Wang W., Krulwich T.A. (2000). Effects of nonpolar mutations in each of the seven *Bacillus subtilis* mrp genes suggest complex interactions among the gene products in support of Na^+^ and alkali but not cholate resistance. J. Bacteriol..

[103] Hellmer J., Teubner A., Zeilinger C. (2003). Conserved arginine and aspartate residues are critical for function of MjNhaP1, a Na^+^/H^+^ antiporter of M. jannaschii. FEBS Lett..

[104] Padan E., Kozachkov L., Herz K., Rimon A. (2009). NhaA crystal structure: Functional-structural insights. J. Exp. Biol..

[105] Calinescu O., Paulino C., Kuhlbrandt W., Fendler K. (2014). Keeping it simple, transport mechanism and pH regulation in Na^+^/H^+^ exchangers. J. Biol. Chem..

[106] Maes M., Rimon A., Kozachkov-Magrisso L., Friedler A., Padan E. (2012). Revealing the ligand binding site of NhaA Na^+^/H^+^ antiporter and its pH dependence. J. Biol. Chem..

[107] Harding M.M. (2002). Metal-ligand geometry relevant to proteins and in proteins: Sodium and potassium. Acta Crystallogr. D Biol. Crystallogr..

[108] Calinescu O., Danner E., Bohm M., Hunte C., Fendler K. (2014). Species differences in bacterial NhaA Na^+^/H^+^ exchangers. FEBS Lett..

[109] Inoue H., Noumi T., Tsuchiya T., Kanazawa H. (1995). Essential aspartic acid residues, Asp-133, Asp-163 and Asp-164, in the transmembrane helices of a Na^+^/H^+^ antiporter (NhaA) from *Escherichia coli*. FEBS Lett..

[110] Tsuboi Y., Inoue H., Nakamura N., Kanazawa H. (2003). Identification of membrane domains of the Na^+^/H^+^ antiporter (NhaA) protein from Helicobacter pylori required for ion transport and pH sensing. J. Biol. Chem..

[111] Kuwabara N., Inoue H., Tsuboi Y., Mitsui K., Matsushita M., Kanazawa H. (2006). Structure-function relationship of the fifth transmembrane domain in the Na^+^/H^+^ antiporter of Helicobacter pylori: Topology and function of the residues, including two consecutive essential aspartate residues. Biochemistry.

[112] Huang Y., Chen W., Dotson D.L., Beckstein O., Shen J. (2016). Mechanism of pH-dependent activation of the sodium-proton antiporter NhaA. Nat. Commun..

[113] Olkhova E., Hunte C., Screpanti E., Padan E., Michel H. (2006). Multiconformation continuum electrostatics analysis of the NhaA Na^+^/H^+^ antiporter of *Escherichia coli* with functional implications. Proc. Natl. Acad. Sci. USA.

[114] Arkin I.T., Xu H., Jensen M.O., Arbely E., Bennett E.R., Bowers K.J., Chow E., Dror R.O., Eastwood M.P., Flitman-Tene R. (2007). Mechanism of Na^+^/H^+^ antiporting. Science.

[115] Olkhova E., Kozachkov L., Padan E., Michel H. (2009). Combined computational and biochemical study reveals the importance of electrostatic interactions between the “pH sensor” and the cation binding site of the sodium/proton antiporter NhaA of *Escherichia coli*. Proteins.

[116] Dwivedi M., Sukenik S., Friedler A., Padan E. (2016). The Ec-NhaA antiporter switches from antagonistic to synergistic antiport upon a single point mutation. Sci. Rep..

[117] Uzdavinys P., Coincon M., Nji E., Ndi M., Winkelmann I., von Ballmoos C., Drew D. (2017). Dissecting the proton transport pathway in electrogenic Na^+^/H^+^ antiporters. Proc. Natl. Acad. Sci. USA.

[118] Patino-Ruiz M., Dwivedi M., Calinescu O., Karabel M., Padan E., Fendler K. (2019). Replacement of Lys-300 with a glutamine in the NhaA Na^+^/H^+^ antiporter of *Escherichia coli* yields a functional electrogenic transporter. J. Biol. Chem..

[119] Henderson J.A., Huang Y., Beckstein O., Shen J. (2020). Alternative proton-binding site and long-distance coupling in *Escherichia coli* sodium-proton antiporter NhaA. Proc. Natl. Acad. Sci. USA.

[120] Galili L., Rothman A., Kozachkov L., Rimon A., Padan E. (2002). Trans membrane domain IV is involved in ion transport activity and pH regulation of the NhaA-Na^+^/H^+^ antiporter of *Escherichia coli*. Biochemistry.

[121] Mondal R., Rimon A., Masrati G., Ben-Tal N., Friedler A., Padan E. (2021). Towards Molecular Understanding of the pH Dependence Characterizing NhaA of Which Structural Fold is Shared by Other Transporters. J. Mol. Biol..

[122] Goswami P., Paulino C., Hizlan D., Vonck J., Yildiz O., Kuhlbrandt W. (2011). Structure of the archaeal Na^+^/H^+^ antiporter NhaP1 and functional role of transmembrane helix 1. EMBO J..

[123] Gerchman Y., Rimon A., Padan E. (1999). A pH-dependent conformational change of NhaA Na(+)/H(+) antiporter of *Escherichia coli* involves loop VIII-IX, plays a role in the pH response of the protein, and is maintained by the pure protein in dodecyl maltoside. J. Biol. Chem..

[124] Tzubery T., Rimon A., Padan E. (2004). Mutation E252C increases drastically the Km value for Na^+^ and causes an alkaline shift of the pH dependence of NhaA Na^+^/H^+^ antiporter of *Escherichia coli*. J. Biol. Chem..

[125] Tzubery T., Rimon A., Padan E. (2008). Structure-based functional study reveals multiple roles of transmembrane segment IX and loop VIII-IX in NhaA Na^+^/H^+^ antiporter of *Escherichia coli* at physiological pH. J. Biol. Chem..

[126] Nakamura T., Fujisaki Y., Enomoto H., Nakayama Y., Takabe T., Yamaguchi N., Uozumi N. (2001). Residue aspartate-147 from the third transmembrane region of Na(+)/H(+) antiporter NhaB of Vibrio alginolyticus plays a role in its activity. J. Bacteriol..

[127] Habibian R., Dzioba J., Barrett J., Galperin M.Y., Loewen P.C., Dibrov P. (2005). Functional analysis of conserved polar residues in Vc-NhaD, Na^+^/H^+^ antiporter of Vibrio cholerae. J. Biol. Chem..

[128] Ostroumov E., Dzioba J., Loewen P.C., Dibrov P. (2002). Asp(344) and Thr(345) are critical for cation exchange mediated by NhaD, Na(+)/H(+) antiporter of Vibrio cholerae. Biochim. Biophys. Acta.

[129] Yang Z., Meng Y., Zhao Q., Cheng B., Xu P., Yang C. (2018). Critical Functions of Region 1-67 and Helix XIII in Retaining the Active Structure of NhaD Antiporter in *Halomonas* sp. Y2. Front. Microbiol..

[130] Meng Y., Yang Z., Cheng B., Nie X., Li S., Yin H., Xu P., Yang C. (2017). Functional Interaction between the N and C Termini of NhaD Antiporters from *Halomonas* sp. Strain Y2. J. Bacteriol..

[131] Cheng B., Meng Y., Cui Y., Li C., Tao F., Yin H., Yang C., Xu P. (2016). Alkaline Response of a Halotolerant Alkaliphilic Halomonas Strain and Functional Diversity of Its Na+(K+)/H+ Antiporters. J. Biol. Chem..

[132] Nie R., Stark S., Symersky J., Kaplan R.S., Lu M. (2017). Structure and function of the divalent anion/Na(+) symporter from Vibrio cholerae and a humanized variant. Nat. Commun..

[133] Jardetzky O. (1966). Simple allosteric model for membrane pumps. Nature.

[134] Kozachkov L., Herz K., Padan E. (2007). Functional and structural interactions of the transmembrane domain X of NhaA, Na^+^/H^+^ antiporter of *Escherichia coli*, at physiological pH. Biochemistry.

[135] Olkhova E., Padan E., Michel H. (2007). The influence of protonation states on the dynamics of the NhaA antiporter from *Escherichia coli*. Biophys. J..

[136] Lentes C.J., Mir S.H., Boehm M., Ganea C., Fendler K., Hunte C. (2014). Molecular characterization of the Na^+^/H^+^-antiporter NhaA from Salmonella Typhimurium. PLoS ONE.

[137] Calinescu O., Dwivedi M., Patino-Ruiz M., Padan E., Fendler K. (2017). Lysine 300 is essential for stability but not for electrogenic transport of the *Escherichia coli* NhaA Na(+)/H(+) antiporter. J. Biol. Chem..

